# A Novelty Detection Approach for Tendons of Prestressed Concrete Bridges Based on a Convolutional Autoencoder and Acceleration Data

**DOI:** 10.3390/s19071633

**Published:** 2019-04-05

**Authors:** Kanghyeok Lee, Seunghoo Jeong, Sung-Han Sim, Do Hyoung Shin

**Affiliations:** 1 Department of Civil Engineering, Inha University, Incheon 22212, Korea; kanghyeok0117@gmail.com; 2 School of Urban and Environmental Engineering, Ulsan National Institute of Science and Technology, Ulsan 44919, Korea; shjeong@unist.ac.kr (S.J.); ssim@unist.ac.kr (S.-H.S.)

**Keywords:** novelty detection, convolutional autoencoder, bridge damage, prestress tendons, PSC bridge

## Abstract

The most important structural element of prestressed concrete (PSC) bridges is the prestressed tendon, and in order to ensure safety of such bridges, it is very important to determine whether the tendon is damaged. However, it is not easy to detect tendon damage in real time. This study proposes a novelty detection approach for damage to the tendons of PSC bridges based on a convolutional autoencoder (CAE). The proposed method employs simulation data from nine accelerometers. The accuracies of CAEs for multi-vehicle are 79.5%–85.8% for 100% and 75% damage severities with all error levels and 50% damage severity without error. However, the accuracies for 50% damage severity with 5% and 10% error levels drop to 69.4%–73.3%. The accuracies of CAEs for single-vehicle ranges from 90.1%–95.1% for all damage severities and error levels that are satisfactory. The findings indicate that the CAE approach for multi-vehicle can be effective when the damages are severe, but not when moderate. Meanwhile, if acceleration data can be obtained for single-vehicle, then the CAE approach can provide a highly accurate and robust method of tendon damage detection in PSC bridges in use, even if the measurement errors are significant.

## 1. Introduction

Prestressed concrete (PSC) bridges have an advantage compared with reinforced concrete bridges in that their structures can withstand large loads even with a small amount of rebar and concrete. PSC bridges are also economical and durable, as they can be used for 75 to 100 years if corrosion of the steel-wire tendon is prevented. Owing to these advantages, PSC bridges are widely used [1].

As the number of aging PSC bridges increases across the world, there have been many reports on damage to tendons owing to corrosion in PSC bridges. Some of these cases were severe and PSC bridges such as the Melle Bridge in Belgium and Shinsuga Bridge in Japan even collapsed owing to tendon corrosion. These cases indicate the importance of detecting tendon damage in PSC bridges to prevent their collapse and ensure safety. In practice, however, it is very difficult to detect early symptoms through bridge inspections before failures of tendons in PSC bridges occur [2].

Recently, structural health monitoring (SHM) has been widely used to monitor the behavior of a bridge and detect its damages through various sensors. However, as the structural behavior of PSC bridges does not change significantly according to damages, difficulties in detecting tendon damages still exist even with the use of SHM. To address this limitation, studies on novelty detection for bridges have been actively conducted to detect damages even with small behavior changes in bridges.

Novelty detection using SHM is based on identifying changes in the response to the undamaged and damaged states to evaluate the state of a bridge. Damage-sensitive features, such as the modal properties, wavelet coefficients, and principal component analysis (PCA), which are calculated from the response of a bridge, are mainly used for the state evaluation. However, even if the bridge damage is considerable, it is usually difficult to detect the damage using damage-sensitive features because of the slight difference. Analytical tools, such as the Mahalanobis squared distance (MSD) and artificial neural network (ANN), have also been used to more clearly distinguish damage-sensitive features based on the state of a bridge.

The MSD is widely used in various industries to identify outliers that significantly deviate from normal data. However, the MSD has a limitation. It considers the importance of all variables to be the same. As a result, this characteristic can lower the accuracy of novelty detection if numerous unimportant and redundant variables are included in the data.

In contrast, the ANN considers the importance of variables and thus is widely used for novelty detection. Most ANN approaches to analyze damage-sensitive features are based on supervised learning, which requires label data as correct answers for an output layer. During novelty detection for a bridge in service, the label data are the actual damages of the bridge and are generally obtained through model updates. However, the process of obtaining data requires time and effort; it is limited in that it is difficult to accurately determine the state of the bridge because it is impractical to conduct a destructive experiment on a bridge in service.

This limitation can be solved by using a convolution autoencoder (CAE), which is an ANN based on unsupervised learning. The CAE uses an encoder network for the extraction of features and a decoder network that restores the input data of the ANN based on the features. Thus, the CAE uses the advantages of an ANN in that it extracts features while not requiring label data from the state measurements of a bridge. In addition, the CAE extracts convolution features from raw response data using the convolutional neural network (CNN), which has the advantage of preventing information loss that may occur in the damage sensitive features. Thus, novelty detection of the tendons of PSC bridges can be more efficiently performed using the CAE.

The purpose of this study is to develop a novelty detection approach for damage to the tendons of PSC bridges using the CAE. Measured response data from bridges with damage to tendons are necessary to verify the performance of the CAE. However, it is practically impossible to obtain actual data by intentionally breaking a bridge tendon because it poses a safety risk to the bridge users. Therefore, simulation data were used for training and testing the CAE instead of actual data. The simulation model was applied to “Hannam 2 Overpass”, which is a PSC-I bridge used in Seoul, Korea. The simulation was performed using the MIDAS/CIVIL program.

## 2. Related Studies

Novelty detection for SHM has attracted attention owing to its ability to identify outliers in signal data of the structural responses of a bridge. Novelty detection approaches mainly have used damage sensitive features from responses to evaluate the structure state.

Most studies on damage-sensitive features have used modal properties such as mode shapes and modal frequencies. Kirmser [3] reported that the cracking of an iron beam affects its natural frequency. Casas and Aparicio [4] proposed approaches to detect the damage of a bridge based on changes of the modal properties; many related studies have been conducted since then. For example, Blachowski et al. [5] recently showed that a high-level modal frequency in 13th mode or above could reflect the damage in a steel frame structure. Soman et al. [6] proposed a damage detection approach using three indexes that are calculated using the natural frequency and mode shapes as damage-sensitive features from displacement, strain, and acceleration and each index was used to identify the damages of four parts of a long-span suspension bridge.

Many studies have been carried out on novelty detection with wavelet coefficients. For example, Hou et al. [7] proposed a novelty detection approach using wavelet analysis that is often used in signal processing. Khatam et al. [8] used wavelet analysis for the novelty detection for beams within harmonic loading. Noh et al. [9] suggested three wavelet-based damage-sensitive features obtained through wavelet analysis. Recently, Pnevmatikos et al. [10] and Pnevmatikos and Hatzigeorgiou [11] proposed novelty detection approaches for steel frame structures using wavelets coefficients as damage-sensitive features.

The features of PCA are also useful for pattern recognition of high-dimensional data using dimension reduction and there are many studies that use PCA for SHM (e.g., [12,13,14,15,16]). Manson [12] developed a feature-based damage detection technique using PCA components that can detect damage while filtering out temperature effects, which have a significant impact on the structure. Gharibnezhad et al. [13] used the robust PCA [17] to solve the problem of sensitive covariance in conventional PCA owing to the anomalies present in the data. The robust PCA showed better performance and faster testing than the conventional PCA for the problem of detecting an outlier in dynamic signal data obtained from seven piezoelectric sensors. Reynders et al. [14] presented a novelty detection approach for three-span concrete bridges using kernel PCA for nonlinear systems affected by changing environmental conditions. Mujica et al. [15] used PCA to determine hidden patterns in the dynamic responses of aero structures. The correlation between PCA components and structural damage was clarified, and PCA obtained from the signal data of the structure showed that anomalies can be used for damage detection. Wah et al. [16] developed a damage detection technique that uses a Gaussian mixture model in PCA and applied it to truss bridges to verify the technique.

Many studies are based on the application of analytical methods such as MSD (e.g., [18,19,20,21,22]) or ANN (e.g., [23,24,25,26,27,28,29,30,31]) to achieve a higher accuracy of novelty detection using damage-sensitive features. The MSD-based method describes the detection of novelty data by setting up a category for normal data and detecting outliers. For example, Worden et al. [18] proposed an MSD method to detect outliers of a normal data category for univariate and multivariate data. Sohn et al. [19] estimated the outliers using the MSD between real strain data and fitted values of an autoregressive model formed using the strain data of a bridge. Mosavi et al. [20] showed that damage localization of a steel beam using MSD is possible when using vector autoregressive model coefficients to generate acceleration data of a simple steel beam structure. Zhou and Wahab [21] developed a faster novelty detection approach using MSD, where the transmissibility of the structures is affected by unknown excitation. Zhou et al. [22] extracted PCA features based on a frequency response function calculated using response data and used it to detect the damage (anomalies) of the structure with MSD. However, the MSD does not consider the importance of each variable and the accuracy may not be ensured.

In contrast, ANN-based methods consider the importance of variables based on features. The ANN-based methods are also useful for pattern recognition in high-dimensional data and has been adopted for SHM. The approaches using ANN mainly use the extracted damage sensitive features from signal data of a structure. For example, Mehrjoo et al. [23] used an ANN for detecting the damage of each node of a truss structure and obtained a damage detection accuracy of approximately 1% when using the first- to fifth-order mode shapes obtained from the signal data as input data for an ANN. Park et al. [24] proposed a damage detection approach using an time-based features and a modal-based features with neural network. They verified the approach for simply supported beams. Shu et al. [25] proposed a damage detection approach that identifies the damage severity and damage location of a simplified railway bridge model based on an ANN. Goh et al. [26] employed ANN to estimate the mode shape, which cannot be measured with a small number of sensors and used the mode shape for novelty detection. Hakim et al. [27] proposed a damage detection approach for a beam structure using an ensemble network consisting of five ANN networks extracted using five mode shapes (first- to fifth-order mode shapes), which were obtained from acceleration data as input data for each ANN. Tan et al. [28] used an ANN to detect single and multiple instances of damage in a simply supported steel I-beam. Padil et al. [29] proposed a non-probabilistic ANN approach for damage detection using modal properties extracted from response data including unknown noises.

During ANN-based novelty detection, more sophisticated neural networks can be used to extract clearer features, thus providing a higher accuracy. However, because ANN is generally based on supervised learning, it is necessary to calculate the loss of the backpropagation algorithm. To calculate the loss, label data are required for the output layer of the ANN. The ANN cannot be trained without label data.

To apply an ANN in a novelty detection approach for an actual bridge, various damage states of a bridge are required as the label data of an ANN. However, determining the damage state of a bridge is difficult because it is impossible to intentionally cause damage to a bridge in service. Therefore, it is necessary to create an analytical model that can provide various damage states of a real bridge. However, as the behavior of an analytical model is generally different from that of an actual structure, a model-updating process is required to make the analytical model similar to a real bridge. The model-updating process requires significant time and effort to construct the analytical model and to update the model with high accuracy.

Some studies used an unsupervised learning-based ANN for novelty detection. For example, Yeung and Smith [30] demonstrated the possibility to detect damages of a bridge with damage-sensitive features using an unsupervised learning-based ANN, but their methods showed that the limit is very sensitive to measurement noises. Figueiredo et al. [31] demonstrated that an unsupervised learning-based ANN provides a better accuracy than other unsupervised learning-based machine learning algorithms such as factor analysis, MSD, and singular value decomposition. These approaches have the advantage of being able to detect damages without a model updating process and label data, although it is not possible to estimate the damage locations.

An autoencoder (AE) proposed by Hinton and Salakhutdinov [32] is considered as an unsupervised learning-based ANN having a more robust performance due to its characteristics. As an AE uses input data as the label data, an AE model is trained in a direction that can restore normal input data well. If normal data are input to the AE model after training, restoration proceeds smoothly, but this is not the case with abnormal data. This makes it possible to determine novelty. Recently, novelty detection using AE has been attracting attention with the progress of deep learning, and many related studies have been conducted in various fields (e.g., [33,34,35,36,37,38]). For example, Shin et al. [33] proposed a multiple organ detection technique for patients based on 4D medical images using a stacked AE. Yan and Yu [34] proposed a technique to detect the novelty of a gas turbine using a stacked denoising AE. Xiong and Zuo [35] used a deep AE to detect geochemical anomalies. Jiang et al. [36] attempted to determine anomalies in chemical sensor data using an active AE, and Oh et al. [37] estimated anomalies in Surface-Mounted Device machine sound data using an AE.

These studies have shown that AEs enable novelty detection with high accuracy in various areas. The results show that AEs can also be used to detect bridge damages. In this study, a novelty detection approach using an AE was developed for PSC bridges. Lin et al. [39] showed that features extracted from raw response data through a convolutional neural network (CNN) are of advantage in novelty detection. Lee et al. [40] also showed that multiple variable data from bridges can be efficiently used with a CNN. Therefore, in this study, a CAE was used, which efficiently utilizes an AE and CNN for response data from bridges.

## 3. Multi-vehicle Traffic Loads

As described in the Introduction section, it is impractical to cause damage to a real bridge and obtain its actual response data. Therefore, this approach was developed using simulation data from an FE model similar to the “Hannam 2 Overpass” in Seoul, Korea. The simulation data was obtained from the base simulations. Each base simulation was performed under the condition that only one vehicle moves in only one lane at a constant speed, with one of two bridge states: undamaged state (without tendon damage) and damaged state (with tendon damage). As a result of each base simulation, the base data consisting of time-series acceleration data from multiple measurement points were obtained.

The Hannam 2 Overpass has four lanes so that several vehicles are frequently on the bridge simultaneously. Therefore, this situation was considered a priority. The acceleration data of the multi-vehicle traffic loads were generated by combining multiple base data based on the additivity of acceleration. The CAE was trained with only the acceleration data of multi-vehicle traffic loads in the undamaged state, whereas its test to identify the state to be either undamaged or damaged was conducted with the acceleration data in both states.

The process of developing the CAE for novelty detection for the tendons is organized as follows: In Section 3.1, the base data are generated with 15 vehicle speeds in each of the four lanes. In Section 3.2, the datasets for the multi-vehicle traffic loads are generated using multiplication and combination processes with base data and divided into training and test datasets. In Section 3.3, the CAE architecture and corresponding hyperparameter are configured. In Section 3.4, the CAE test results are discussed.

### 3.1. Base Data Generation

**Figure 1:**
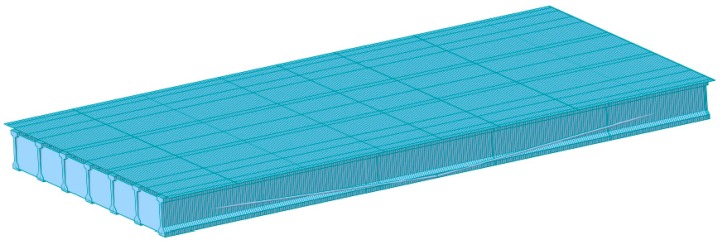
PSC I-girder bridge model with external tendon at both ends of girder.

A linear elastic FE model of a single-span PSC-I girder of the Hannam 2 Overpass was built to generate acceleration responses before and after the damage of the external tendon using the structural software MIDAS/CIVIL (Figure 1). The bridge model is supported at the ends by hinge and roller supports with a span of 28.35 m and width of 13.2 m. The FE model has four lanes and two-way traffic and consists of a bridge deck modelled by plate elements, and seven PSC I-girders, six cross beams, and four external tendons modelled by beam elements. The bridge components, girder, deck, and cross beam were assembled by rigid link. Each PSC I-girder was modeled to have a prestress tension of 10,000 kN. The external tendons were modeled after a Korean standard (KSCE-LSD 15) steel, which was added to the first and seventh girders with a prestress tensile load of 80,000 kN. It was verified that the deck of the FE model was in compression due to the prestress tension. The detailed material and sectional properties of each component are shown in Table 1.

**Figure 2:**
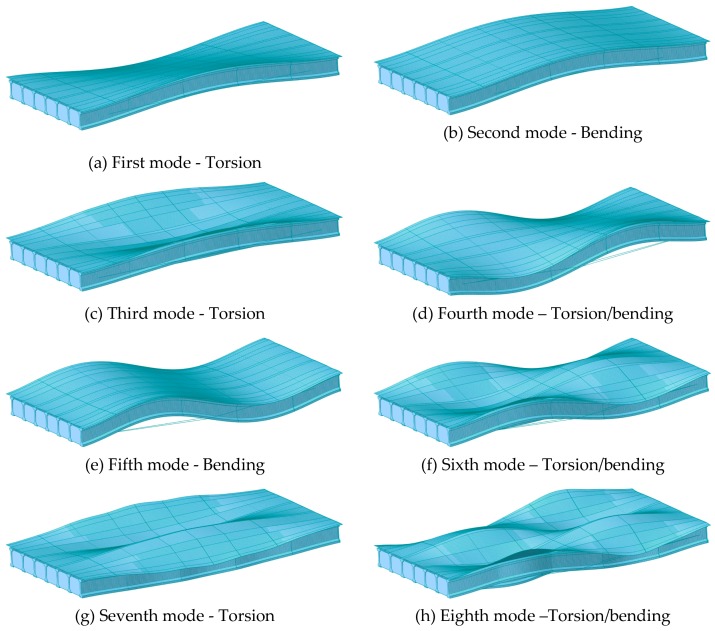
Mode shapes of modeled bridge (undamaged case).

The damaged states of the bridge were modelled by reducing the prestress tension of four external tendons that are attached to the first and seventh girders. Based on the intact external tendons with 80,000 kN tension, this study assumed that 100% loss of tension corresponds to 100% damage state and 75% and 50% loss of tension are identical to 75% and 50% damage severities, respectively. The first eight natural frequencies of the bridge with respect to the damage severities are shown in Table 2. The corresponding mode shapes are shown in Figure 2. It is verified that the FE model had different natural frequencies depending on the damage severity of the external tendons, and internal girder was in compression due to the prestress tension.

Table 2 shows that the bridge with more damage in tendons is likely to have higher natural frequencies owing to geometric change due to the damage in the external tendon. From the previous study, the bridge deflected by the dead load tends to have higher natural frequencies than the bridge without deflection. In this study, the damage in the external tendon deflects the bridge that results in increased natural frequencies as compared to the intact bridge.

**Figure 3:**
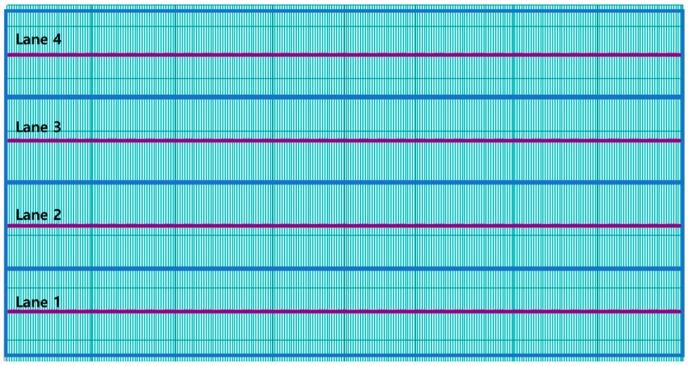
Traffic load points (Lanes 1, 2: right direction, Lanes 3, 4: left direction).

**Figure 4:**
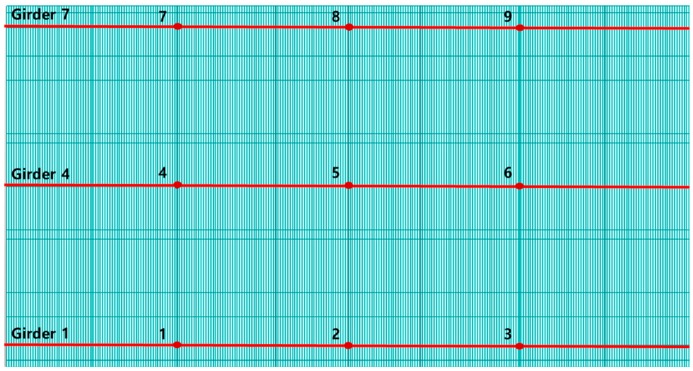
Acceleration measurement points on the bridge deck.

The traffic loads of a single vehicle in each lane were used to simulate a set of acceleration responses of the bridge. Traffic loads for the base data are assumed to be point loads of the front (9 kN) and back (6 kN) axles on each lane, with 10 cm intervals and 15 vehicle speeds (20, 25, 30, 35, 40, 45, 50, 55, 60, 65, 70, 75, 80, 85, and 90 km/h; Figure 3). Note that the mass of a sedan car in Korea is about 1,500 kg, which is equivalent to 15 kN in weight. The acceleration responses under the car loads were measured with a sampling rate of 1,000 Hz for 10 s at nine points on the bridge deck, as shown in Figure 4. The measurement points are located on the quad-, middle-, and third quad-span of the first, fourth, and seventh girders. A linear time history analysis was conducted to generate the dynamic responses of traffic loads at 1,000 Hz using the Runge–Kutta–Fehlberg numerical analysis method.

**Figure 5:**
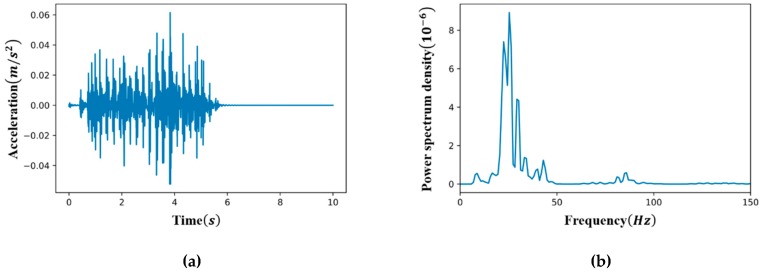
Example of an acceleration data from measurement point 3 when a car passes through lane 1 at 20 km/h (sampling rate = 1,000 Hz) and a power spectral density of acceleration. (**a**) Acceleration data; (**b**) Power spectral density of the acceleration.

The linear time history was analyzed to produce 60 sets of base data of acceleration responses from each of the four damage severities (undamaged, 50%, 75 %, and 100 % damage) for the 15 vehicle speeds in four lanes. A typical example of the acceleration is shown in Figure 5a, which was measured from the 100% damaged structure at measurement point 3 in Figure 4 when a car passes through lane 1 at a speed of 20 km/h. All simulated acceleration signals sampled with 1,000 Hz were downsampled to 70 Hz, which was selected considering the computational efficiency in training the CAE, as well as the first seven natural modes with high energy, as shown in Figure 5b. Note that the anti-aliasing filter of an order 8 type-1 Chebyshev was used before downsampling.

### 3.2. Data Sets

After downsampling, the base data were modified through a multiplication process based on the homogeneity of acceleration, to apply various magnitudes of loads from a vehicle. The magnitude of load of a vehicle that can ply on the bridge was set to be 0.5, 1, or 1.5 times larger than the load of the base data, considering the weights of sedans, small city cars, and vans in Korea. A multiplication process based on the homogeneity of the acceleration data was designed to have the same effect as loading a vehicle with different load sizes. For example, the acceleration data of a load that is 1.5 times as large as the load of the base data can be obtained by multiplying the acceleration data of the base data by 1.5.

**Figure 6:**
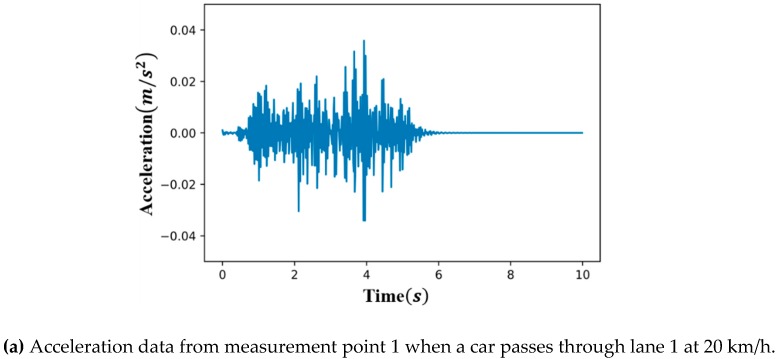

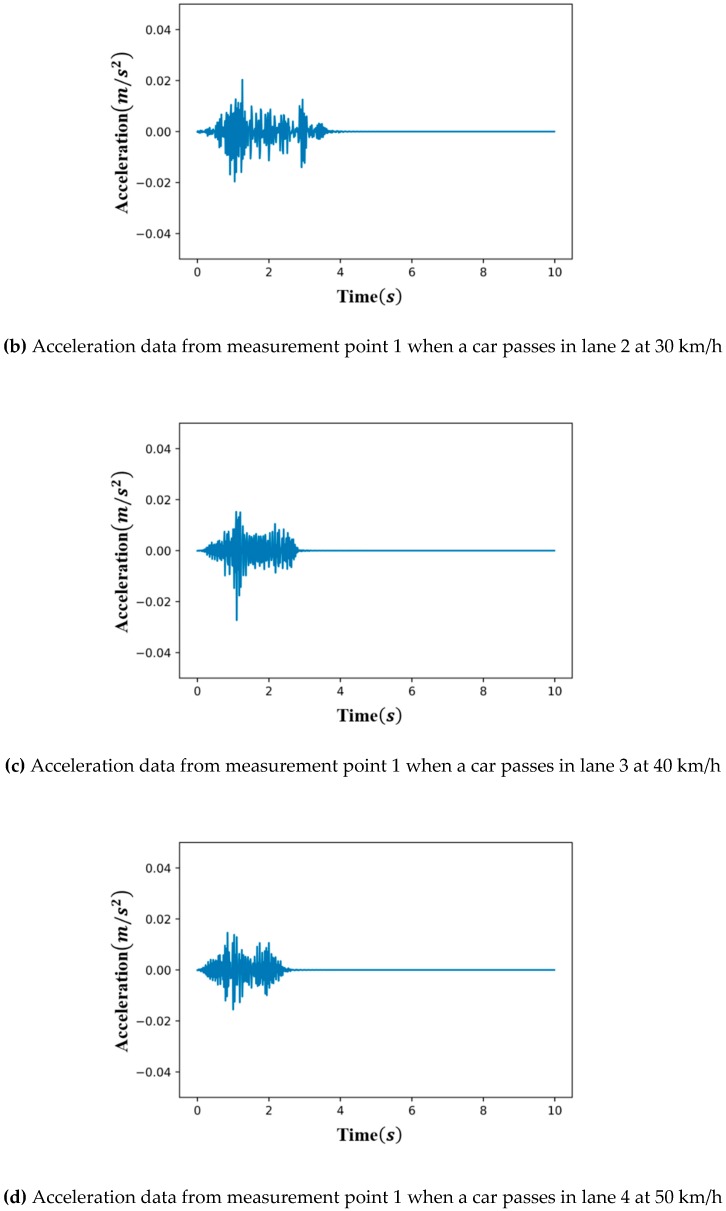

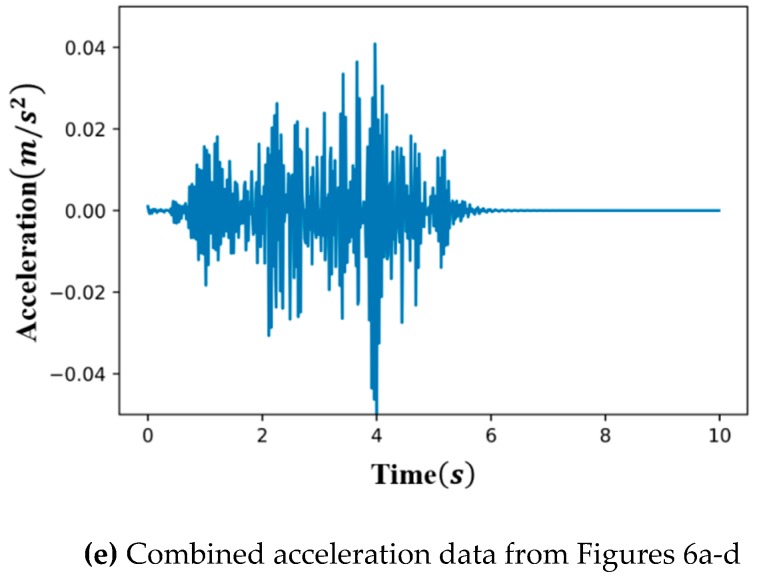
Example of the combination of base data.

To detect the damaged state of the bridge with multi-vehicle traffic loads, the simulation data for the moving vehicles randomly loaded over all lanes were generated by combining the modified base data. Data are combined depending on the additivity of acceleration, as shown in an example in Figure 6. Figure 6a–d show the base data from measurement point 1 in the cases where a car plies at 20 km/h on lane 1, at 30 km/h on lane 2, at 40 km/h on lane 3, and at 50 km/h on lane 4, respectively, with a starting interval of 1 second in the order of lane 1 to lane 4. Figure 6e shows the combined acceleration of the four sets of base data in Figure 6a–d.

During CAE development, the CAE was intended to be trained with varying speeds. It may be strange that vehicles moving at very different speeds travel on the bridge immediately one after another. However, although such situations are rare, they can happen. If the CAE was trained without a dataset for those situations, it could mistakenly detect a damaged status in such situations. Meanwhile, it is unlikely that a vehicle plying at any speed is followed by another vehicle at an entirely different speed in the same lane in practice. The loading protocol for multi-vehicle traffic was designed to comply with this constraint.

The time lag between the appearances of vehicles in the same lane of the bridge was set such that each succeeding vehicle randomly departed 0–3 s after the preceding vehicle left the lane. This time lag was designed to demonstrate various vehicle appearances in the same lane while avoiding the collision between the preceding and succeeding vehicles. The maximum speed was set to 90 km/h and the minimum speed was 20 km/h. In addition, the speed of the succeeding vehicle could be ± 10 km/h than the preceding vehicle. For example, if the speed of the preceding vehicle in a lane is 85 km/h, the speed of the succeeding vehicle could be between 75 and 90 km/h. This speed constraint between the succeeding and preceding vehicles was applied to the same lane, and different lanes had different speeds.

**Figure 7:**
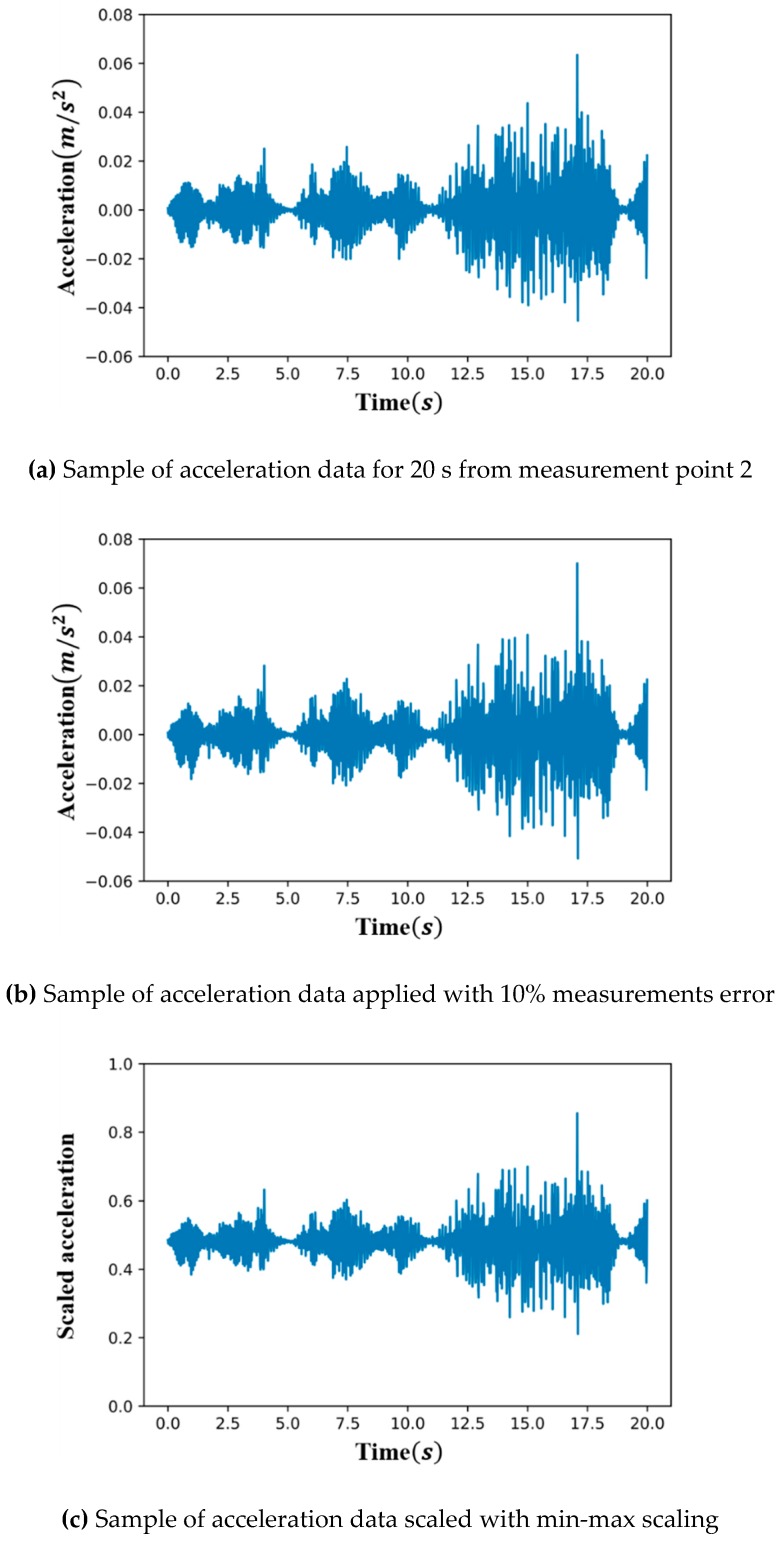
Example of the process for applying the measurements error and min-max scaling.

Based on the loading protocol for multi-vehicle traffic, up to four vehicles should be on the bridge at a given time, while only up to one vehicle should be on each lane. In this manner, the moving vehicles were randomly loaded over all lanes for approximately three weeks (500 h), and the acceleration data was generated by combining the corresponding base data. Figure 7a shows a sample result generated through the data combining process.

In practice, actual measurement data from bridges generally can have measurement errors such as systemic error, instrument error, and installation error, depending on the installation environment. Thus, in this study, random measurement errors were intentionally added to the acceleration data used for the input and output layers of the CAE as in real circumstances.

The random measurement errors were generated using two Gaussian distributions representing different error levels: The mean of all Gaussian distributions was set to 0, and the standard deviations were set as 5% and 10%. The level of measurement errors (or error level) in this study represents the standard deviation of a Gaussian distribution, from where an error for each data point is generated. The random number generated from the Gaussian distribution is used as the percentage of measurement error (noise) of the data point.

For example, in order to apply the error level of 5% to 1,400 data points measured at 70 Hz for 20 seconds, it is necessary to generate 1,400 random numbers from the Gaussian distribution with a standard deviation of 5%. Table 3 shows an example applying the error level of 5% to 1,400 data points. If the random numbers for the first and second data points are 0.0134 and -0.0155, respectively, the acceleration values of the first and second data points are manipulated from 0.0477 m/s^2^ to 0.0483 m/s^2^ with an error of 1.34%, from 0.0482 m/s^2^ to 0.0475 m/s^2^, with an error of -1.55%, respectively.

Three levels of measurement errors, including no error, were set and applied to the 125-hour data. For each level of measurement errors, the training and test sets were revised such that the error for each data point in the training or test sets is randomly generated within the corresponding Gaussian distribution. As a result, a training set, test set, and CAE were generated for each of the three levels of measurement errors. Figure 7b shows the sample of acceleration data with a 10% measurement error.

Neural networks, including CAE, are characterized by good learning when the variations of the input variables are similar. Thus, for effective learning, the acceleration data of each of the nine sensors were scaled using the maximum and minimum data points of the respective sensor for 500 h. This scaling method was applied to the undamaged and damaged states, respectively. Figure 7c shows the sample of scaled acceleration data based on the data in Figure 7b.

**Figure 8:**
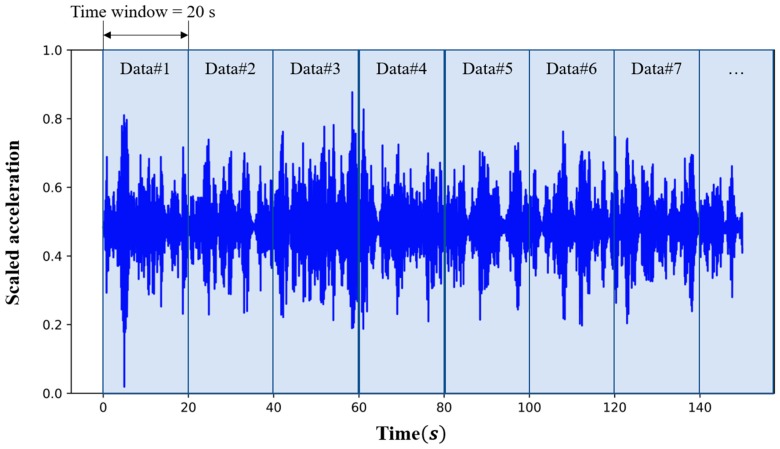
Concept of the time window of the acceleration data (This graph shows a sample of 125-hour acceleration data; the three dots indicate that there are many data in the applied time window).

As shown in Figure 8, the patterns of the behavior in a bridge did not appear well when the time window of the acceleration data is too short. In order to identify the patterns more clearly, the time window for the acceleration data was determined to 20 s (1,400 frames). For this approach, data can be obtained from nine acceleration sensors (see Figure 4) and the sampling rate is 70 Hz. The matrix of the acceleration data for 20 s designed to be of size 9 (the number of sensors) × 1400 (= 20 s × 70 Hz).

Because the combination process to generate the data set requires a lot of time, the combination process for the undamaged state was performed together with those for the three damaged states for temporal efficiency. A total of 22,500 data for 20 s were obtained by dividing time-series acceleration data over approximately five days (125 hours) by the time window (see Figure 8) in the undamaged state and each damaged state, respectively. The training set for the CAE consisted of 15,750 data in the undamaged state, i.e., 70% of all the data in undamaged state. Considering the loading protocol explained above, the size of the training set is considered sufficient to cover most cases of multi-vehicle traffic loads. The other 30% of the data (6,750 data) in undamaged state were used in the test set. The test set for each damaged state also requires data in the damaged state to obtain the detection accuracy for the CAE. Thus, 6,750 data among the 22,500 data in the damaged state were randomly selected and added to the test set, thus balanced with the data in the undamaged state. Thus, the final test for each damaged state set had 13,500 data. The process for configuring the training set and the test sets was conducted for each measurement error.

### 3.3. CAE Architecture

**Figure 9:**
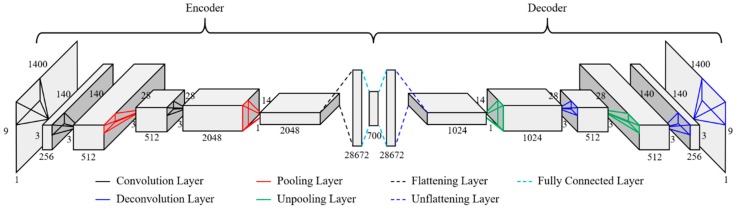
CAE architecture for multi-vehicle traffic loads.

Based on the matrix size of the previously designed data, the architecture for CAEs for novelty detection of bridges with multi-vehicle traffic loads is shown in Figure 9. Generally, the architecture of a CAE consists of two CNN architectures composed of an encoder, which generates the features, and a decoder, which restores the original input data (see Figure 9). The same 9 × 1400 acceleration matrix data were used for both the input and output layers of the CAE, but the information about the vehicle load was not used. The encoder of this CNN architecture has three convolutional layers, two max pooling layers, and one fully connected layer. The decoder has three deconvolutional layers, two unpooling layers, and one fully connected layer. The number of latent variables (features) is 700, which is 1/18 of the input data.

To improve the accuracy of the CAE, it is important to select and use appropriate hyperparameters during training. In this study, epochs (one epoch represents one pass of the full training set) were set up when the accuracy of CAE for each damage severity and each measurement error showed the best results. The other hyperparameters for CAE training were set to be constant. The batch size was set to 256. Xavier initialization was used as the initialization method and RMSprop was used as the gradient method. The learning and decay rates were set to 0.001 and 0.01, respectively. Because the rectified linear unit (ReLU) and hyperbaric tangent (tanh) in this study was empirically demonstrated to be superior in performance as an activation function, each training process was performed twice independently applying each activation function. The CAE model needs to be overfitted in the training set if its purpose is to cluster two states of data using the CAE loss that represents the difference between actual outputs and predicted outputs. In the study, the mean squared error was used for the function of CAE loss. Because the input data of CAE has no unit by min-max scaling, the CAE losses, too, have no unit. The architecture and hyperparameters of the CAE were designed and configured using Keras (deep learning library) with Python and all training and test processes were performed in the following hardware environment: Intel i7-8700K CPU, 32 GB DDR4 RAM, and two NVIDIA GTX-1080Ti GPUs.

### 3.4. Results and Discussion

**Figure 10:**
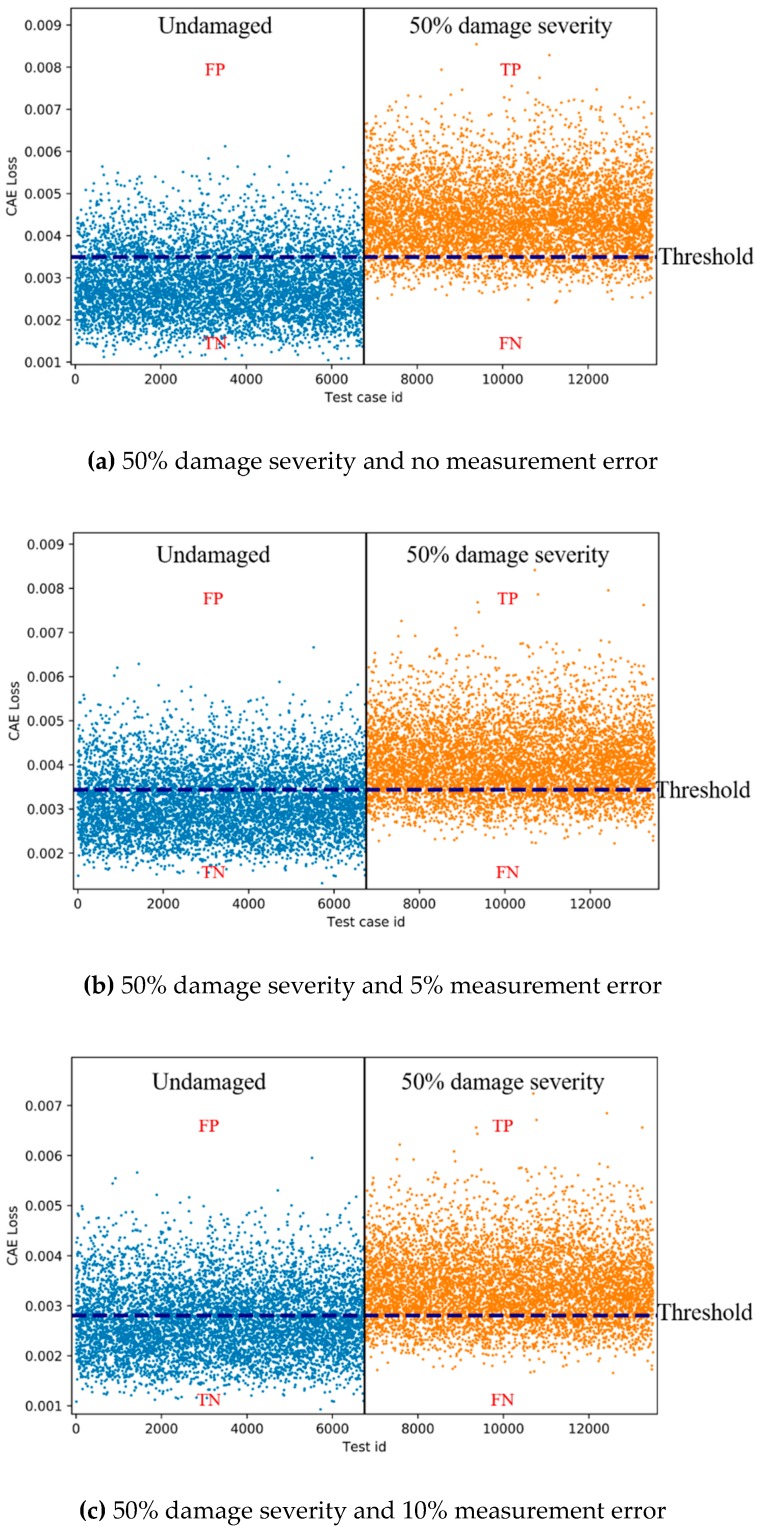
Examples of CAE losses of test data from both undamaged and damaged states for multi-vehicle traffic loads (TP indicates true and positive. FP indicates false and positive. TN indicates true and negative. FN indicates false and negative. True means that the CAE correctly detected the state of bridge. False means that the CAE incorrectly detected the state of bridge. Positive means that the predicted state is the damaged state. Negative means that the predicted state is the undamaged state.).

For each of the three measurement errors, the CAE was trained and tested using the corresponding training and test data sets. Table 3 shows the best accuracies of CAE for the three measurement errors and the corresponding false negative rates (FNRs), false positive rates (FPRs), epochs, activation functions, final training losses and thresholds. Figure 10 shows the examples of CAE losses of the test data (with 50% damage severity) from both the undamaged and damaged states for multi-vehicle traffic loads. As shown in Figure 10, the CAE needs a baseline threshold to cluster the two states of the bridge. There is no unit in the thresholds as in the CAE losses. The thresholds were set with the trial-and-error approach to ensure the best accuracies.

The accuracy calculated by Equation (1) represents the ratio of the total number of true positive (TP) and true negative (TN) sets to the number of total test sets (Figure 10), indicating the rate at which the CAE correctly clusters the test set. The final training loss is the training loss of the final epoch, and its value close to zero means that the CAE is well trained:

(1) $$\mathrm{Accuracy}\;=\frac{TP+TN}{TP+TN+FP+FN}$$

As shown in Table 3, the accuracies for 100% damage severity are 81.0%–82.1% when there is no measurement error or a 5% or 10% measurement error. The accuracies for 75% and 50% damage severities are 79.5%–85.8% and 69.4%-84.0%, respectively, when there is no measurement error or a 5% or 10% measurement error. These results indicate that the accuracies of CAEs are not high enough, but are still acceptable for 100% and 75% damage severities with all error levels. However, the results show unsatisfactory accuracy for 50% damage severity with the error levels of 5% and 10%, while its accuracy with no error is satisfactory. The difference in the accuracies for 50% damage severity between no error and 5% and 10% error levels is also supported by the scatter plots of the CAE losses (which is a measure of how far the predicted CAEs are from the actual value) of the test data for 50% damage severity in Figure 10. While the distinction between the undamaged and damaged states is moderately apparent with no error as shown in Figure 10a, the distinctions are vague with 5% and 10% error levels as shown in Figure 10b,c. For the performance verification of the CAEs, besides the accuracies, false negative (FN) should be considered. FN is a wrong negative test result that occurs when a CAE incorrectly predicts the damaged state of a bridge. Since FN might lead to loss of life and collapse of the bridge, FN is more severe than false positive (FP) that is a false alarm. Therefore, with the best accuracy thresholds, the FNRs calculated by Equation (2) need to be checked carefully. The results shown in Table 3 confirm that the FNRs for multi-vehicle traffic loads are relatively low (5.3%–8.3%) when the accuracies are acceptable (79.5%–85.8%). The FNRs are also 1.5–2.1 times lower than the corresponding FPRs calculated by Equation (3). These results show that the FNRs remain low with the best accuracy thresholds:

(2) $$\mathrm{False}\;\mathrm{negative}\;\mathrm{rate}\;\left(\mathrm{FNR}\right)=\frac{FN}{TP+TN+FP+FN}$$

(3) $$\mathrm{False}\;\mathrm{positive}\;\mathrm{rate}\;\left(\mathrm{FPR}\right)=\frac{FP}{TP+TN+FP+FN}$$

**Figure 11:**
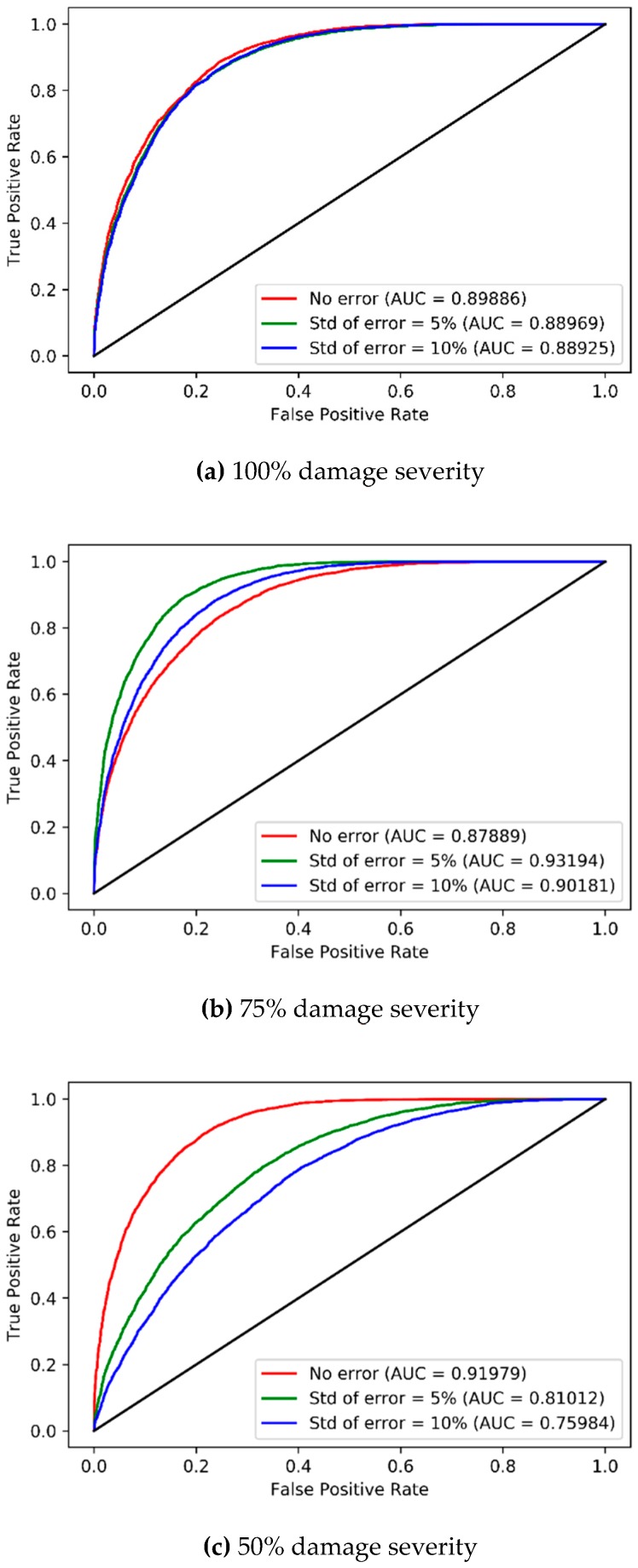
ROC curves and AUCs of CAE models corresponding to three levels of measurement errors for multi-vehicle traffic loads.

The results of accuracies of the CAEs can be confirmed based on the area under the curve (AUC) of the receiver operating characteristic (ROC), as shown in Figure 11. The AUC indicates the classification ability of the classifiers. The AUC ranges of the developed CAE for 100% damage severity and 75% damage severity are 0.89–0.90 (Figure 11a) and 0.88–0.93 (Figure 11b) for different levels of measurement errors, respectively. The AUCs for 50% damage severity are 0.92 with no error, 0.81 with 5% error level, and 0.76 with 10% error level, respectively (Figure 11c). The AUCs remain near 0.9 for 100% and 75% severities with all error levels, indicating good classification ability, and for 50% severity with no error. However, the AUCs drop to 0.8, or less for 50% severity with 5% and 10% error levels. These results show that the CAEs can accurately classify undamaged and damaged states for 100% and 75% damage severities with error, but the CAEs will not be robust and can fail to classify the states with good accuracy for 50% damage severity with measurement errors. Therefore, it is concluded that the CAEs for multi-vehicle traffic loads are effective when damages are severe, but not when damages are moderate.

Considering that the accuracy and AUC are acceptable for 50% damage severity with no error, the low accuracies and AUCs for 50% severity with 5% and 10% error levels are probably because the difference in the patterns of acceleration data between undamaged and damaged states become unclear amid errors. This limitation can be addressed to use acceleration data with clearer patterns. As shown in Figure 6, a single-vehicle traffic load (only one vehicle is on the bridge at a given time) generates clearer patterns of acceleration data compared to multi-vehicle traffic loads. Therefore, it is considered that the undamaged and damaged states of the bridge can be better distinguished by using acceleration data for a single-vehicle traffic load for the development of CAEs.

## 4. Single-vehicle Traffic Load

The CAE development process for single-vehicle traffic load is the same as that with multi-vehicle traffic loads, but the acceleration data from single-vehicle traffic load were used for training and testing the developed CAE. The CAE was trained using the data set of undamaged state only, and the trained CAE was validated with the test data from both undamaged and damaged states as in the case of the multi-vehicle traffic loads.

The process of developing the CAE for a single-vehicle traffic load case is organized as follows: in Section 4.1, the datasets from the single-vehicle traffic load are generated using a multiplication process with base data and divided into training and test datasets. In Section 4.2, the architecture and corresponding hyperparameter for the CAE are configured. In Section 4.3, the test results of the CAE are discussed.

### 4.1. Data Sets

In the case of a single-vehicle traffic load, it is possible to use only 60 sets of base data for each of the four damage severities (undamaged, 50%, 75%, and 100% damage), as described in Section 3.1 because the base data are simulated with a single-vehicle traffic load on the Hannam 2 Overpass. However, the amount of base data is too small for training and testing. Therefore, a multiplication process was employed to increase the amount of data. Consequently, 1,260 data were generated in the undamaged state and the damaged states, respectively for loads that are 0.5–1.5 (by 0.05 increments) times larger than the load of the base data.

However, it takes up to 6 s for the vehicle with the slowest speed (20 km/h) to pass through the bridge. Accordingly, the time window of the data was set to 10 s, and the sampling rate was 70 Hz (see Section 3.1). There are nine sensors; thus, the matrix of each data for 10 s is designed to be of size 9 (number of sensors) × 700 (= 10 s × 70 Hz).

The ratio of training set to test set is the same as that in the preceding multi-vehicle traffic loads’ case. The training set for CAE consisted of 882 data in only the undamaged state, which were 70% of all the data (1,260 data) in the undamaged state. The test set consisted of both the remaining 30% data (378 data) in the undamaged state and 378 data (randomly selected among 1,260 data) in the damaged state to balance the data from the undamaged state. Thus, the final test set had 756 data. Moreover, random measurement errors were intentionally applied to the acceleration data as in the case of multi-vehicle traffic loads.

The measurement errors applied to the acceleration data are based on Gaussian distribution in the same way they were used for multi-vehicle loads: the mean of all Gaussian distributions is zero and the standard deviations are set to 5% and 10%. Thus, three levels of measurement errors were set, including no error, and each measurement error was applied to the training and test sets. Finally, a training set, test set, and CAE were generated for each of the three levels of measurement errors.

### 4.2. CAE Architecture

**Figure 12:**
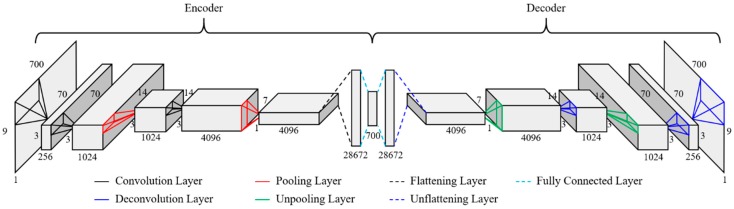
CAE architecture for single-vehicle traffic-load case.

Figure 12 shows the CAE architecture for the single-vehicle traffic load. The acceleration data with a matrix of size 9 × 700 were used for both the input and output layers of the CAE architecture, and any information about the vehicle load was not used. The CAE architecture uses the same number of layers and, correspondingly, the same size of filters. The input matrix size of the single-vehicle traffic load is smaller than the input matrix size of the multi-vehicle traffic loads. Thus, faster training and testing are possible.

The hyperparameters (epoch, regularizer, initialization, learning rate, activation function, etc.) were set for CAE training in the same way as for the multi-vehicle traffic loads. However, as the architecture is smaller than that in case of the multi-vehicle traffic loads, faster training can be achieved in the single-vehicle traffic load case. Based on the designed architecture and hyperparameters, each training set with different error levels was trained. This training process was performed for each of the three error states. The training and tests of the single-vehicle traffic load were performed with the same software and hardware environments that were used for the multi-vehicle traffic loads.

### 4.3. Results and Discussion

The CAEs were trained using each training set with three levels of measurement errors and tested using the corresponding test set. Table 4 shows the best accuracies for the three levels of measurement errors and three damage severities of the CAEs and corresponding FNRs, FPRs, activation functions, epochs, final training losses and thresholds. As in multi-vehicle traffic loads, the thresholds for single-vehicle traffic load were set with the trial-and-error approach to ensure the best accuracies. The accuracies for 100% damage severity are 91.9%–95.1% when the measurement error is 0% (no error), 5%, or 10%. The accuracies for damage severities of 75% and 50% for the three measurement errors are 90.1%–92.6% and 91.9%–92.5%, respectively. The accuracies for single-vehicle traffic load are satisfactory for all damage severities. The measurement errors do not show any significant effects on the accuracies for any damage severity, thereby enabling a robust CAE. For single-vehicle traffic load, as shown in Table 4, the low value of FNRs (0–0.5%) was also confirmed. From these results, for similar to multi-vehicle traffic loads, it can be concluded that the FNRs remain low with the best accuracy thresholds.

**Figure 13:**
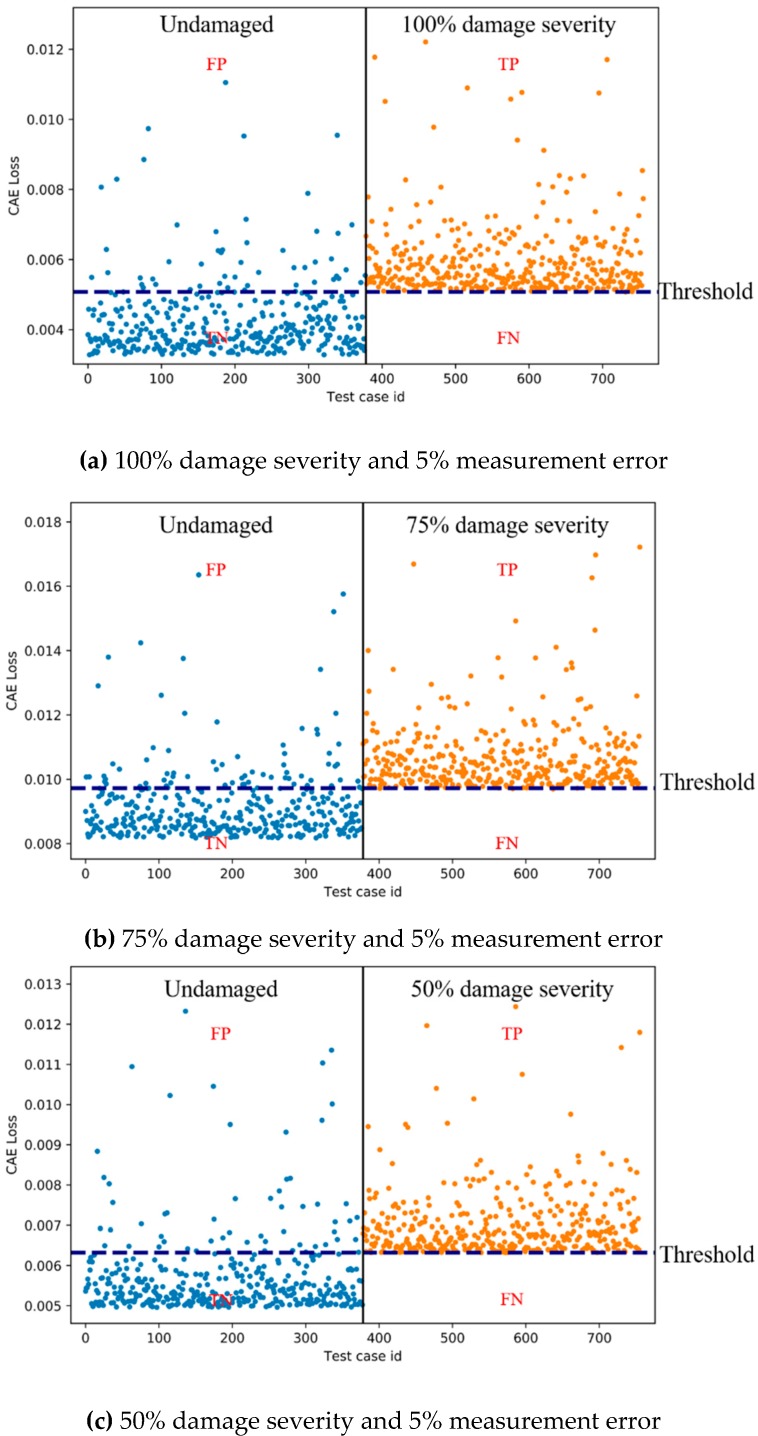
Examples of CAE losses of test data from both undamaged and damaged states for single-vehicle traffic load (TP indicates true and positive. FP indicates false and positive. TN indicates true and negative. FN indicates false and negative. True means that the CAE correctly detected the state of bridge. False means that the CAE incorrectly detected the state of bridge. Positive means that the predicted state is the damaged state. Negative means that the predicted state is the undamaged state).

**Figure 14:**
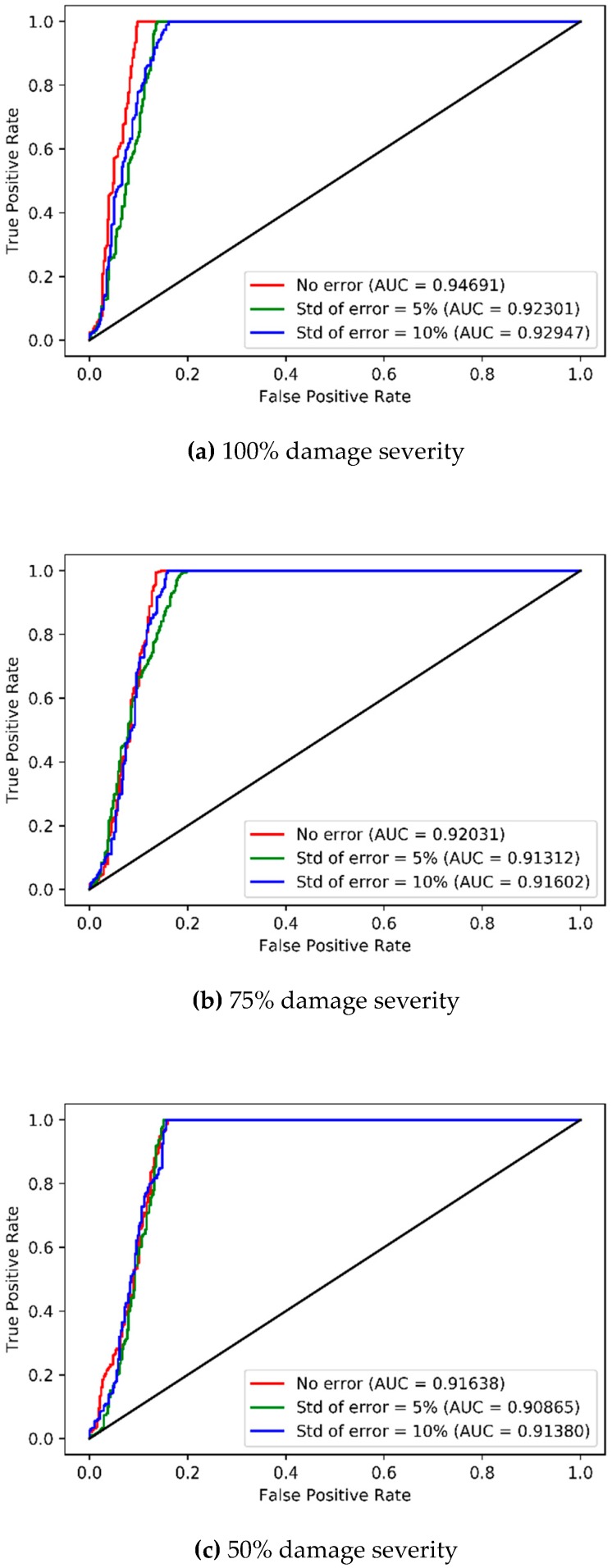
ROC curves and AUCs of CAE models corresponding to three levels of measurement errors for single-vehicle traffic load

The high accuracies of CAEs for single-vehicle traffic are proved in Figure 13 and Figure 14. In Figure 13, the scatter plots of the CAE losses in the test data reveal an apparent distinction between the undamaged and damaged states. In addition, as shown in Figure 14, the AUCs of the ROC curves for 100%, 75%, and 50% damage severities are 0.92–0.95, 0.91–0.92, and 0.91–0.92, respectively, which indicates excellent classification ability. These results demonstrate the robust classification ability and satisfactory accuracy of the CAE.

**Figure 15:**
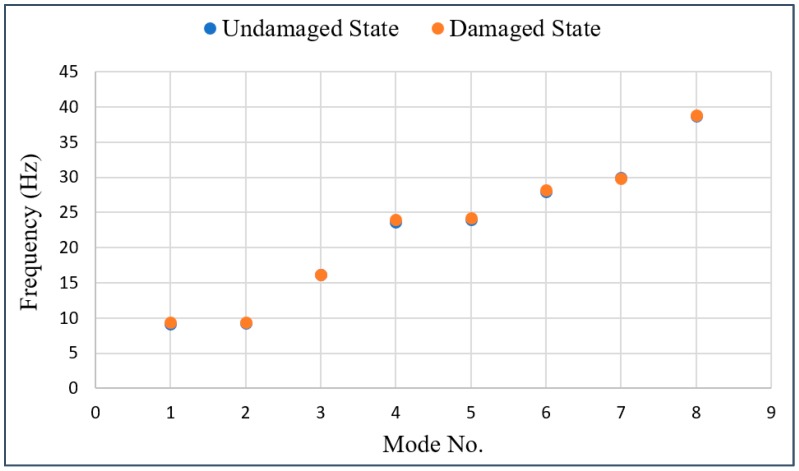
Natural frequencies in modes for both undamaged and damaged states of the Hannam 2 Overpass.

These results also show that the CAE-based damage detection approach can provide a more useful feature than the damage sensitive features, such as the natural frequencies in modes calculated using the acceleration data of the bridge. For example, Figure 15 shows the natural frequencies in modes 1 through 8 obtained by simulating undamaged and damaged bridges without any measurement error. As shown in Figure 15, comparing the damage of the tendon with these natural frequencies in modes would be difficult because the average difference of the frequencies, according to each state of the bridge, is only up to 1%.

Because the CAEs for single-vehicle traffic load can provide satisfactory and robust accuracies even with measurement errors, it will be possible to apply the CAEs to real bridges where measurement errors are frequent. As single-vehicle traffic load can frequently occur early in the morning or late at night, it should be possible to acquire acceleration measurement data from single-vehicle traffic load. Therefore, the CAE-based novelty detection approach for single-vehicle traffic load can be applied to detect tendon damages in PSC bridges in use, regardless of the damage severity.

However, the threshold setting would remain an issue while applying the CAEs in practice because the damage severities and measurement errors are unknown. An alternative to set appropriate thresholds is to use the CAE losses from undamaged states. The results of this study demonstrated the following: for multi-vehicle traffic loads, the values of the third quartile (3Q) of the CAE losses from the undamaged states are close to the best accuracy thresholds. For single-vehicle traffic load, the values of 3Q + 0.3 interquartile range (IQR) of the CAE losses from the undamaged states are close to the best accuracy thresholds. It is confirmed that the accuracies were slightly compromised even with the approximate thresholds, as shown in Table 5. Even though further study on this topic is needed to implement this approach to the approximate thresholds of bridges of different types and sizes, these findings indicate the feasibility to set appropriate thresholds in practice.

## 5. Conclusions

The purpose of this study was to develop a CAE-based novelty detection approach for tendon damage in a PSC bridge. To train the CAE, data from before and after the damage of an actual bridge are required. However, it is practically impossible to cause damage to a bridge in service intentionally because it poses a safety problem. Therefore, simulation data were used for training and testing the CAE, and a structural model was designed based on the Hannam 2 Overpass. For this approach, acceleration data were generated through a simulation and used for the input and output layers of the CAE. Random measurement errors were also intentionally applied to the acceleration data to make them more realistic.

The accuracies of the CAE test results for multi-vehicle traffic loads are 79.5%–85.8% for 100% and 75% damage severities with all error levels, and 50% damage severity with no error. However, the accuracies for 50% damage severity with 5% and 10% error levels drop to 69.4%–73.3%. These results show that the CAEs can accurately classify undamaged and damaged states for 100% and 70% damage severities even with some error levels, but the CAEs will not be robust and can fail to accurately classify the states for 50% damage severity with measurement errors. The reason for these unsatisfactory accuracies is assumed as the lack of clear patterns of acceleration data due to measurement errors.

However, the acceleration data obtained for the single-vehicle traffic load are much simpler and clearer. Hence, the results were used to develop another approach to harness single-vehicle traffic load. The accuracies of the CAE test results for single-vehicle traffic load are between 90.1% and 95.1%, which are satisfactory for all damage severities and error levels. The measurement errors do not show any significant effect on the accuracies for any damage severity, thus enabling a robust CAE.

Based on the results, it is concluded that the proposed CAE approach for multi-vehicle traffic loads can be effective when damages are severe, but not when damages are moderate. Meanwhile, if the acceleration data can be obtained for a single-vehicle traffic load, the proposed CAE approach can provide a highly accurate and robust method of tendon damage detection in PSC bridges in use, even if the measurement errors are significant.

In practice, environmental conditions vary widely. Therefore, varying conditions must be considered for the practical application of the CAE. However, if these environmental conditions are reflected in the simulation, the simulation time will exponentially increase and it will take too much time to generate the data. Therefore, environmental conditions were excluded from this study; instead we focused on the dynamic behavior of the bridge according to the vehicle loads. Environmental conditions generally cause systematic errors. Based on the AE mechanism, the training set is also biased if an AE is trained with a training set with a systematic error. Therefore, if an AE is trained this way and is tested using a test set with a systematic error, the affection of bias is likely to be excluded. The studies on the CAEs considering the environmental conditions will be carried out in future work.

In addition, for the practical application of the CAEs, the threshold setting would remain an issue because the damage severities and measurement errors are unknown. The findings of this study indicate the feasibility to set appropriate thresholds in practice based on the CAE losses from the undamaged state. Further studies are needed to apply this approach to the approximate thresholds of bridges of different types and sizes. Finally, this study focuses on detecting severe tendon damages because detecting them is difficult. However, developing CAEs that can detect moderate tendon damages for a more effective SHM is necessary. This issue can be further addressed based on this study.

## Figures and Tables

**Table 1:** Material and sectional properties of the bridge components.

Component (Material)	Modulus of Elasticity (MPa)	Poisson’s Ratio	Weight Density (N/m^3^)	Section (mm)	
Bridge deck (Concrete)	$2.70\times 10^{4}$	0.18	$2.35\times 10^{4}$	Plate	Thick plate element
PSC I-girder (Concrete)	$3.09\times 10^{4}$	0.18	$2.35\times 10^{4}$	Beam (PSC-I)	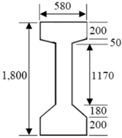
Cross beam (Concrete)	$2.70\times 10^{4}$	0.18	$2.35\times 10^{4}$	Beam (Solid rectangle)	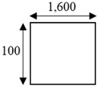
Tendon (Steel)	$2.0\times 10^{5}$	0.3	$7.70\times 10^{4}$	Beam (Solid round)	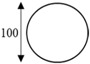

**Table 2:** Natural frequencies (Hz) of modelled bridge depending on the damage severity.

Damage Severity	Natural Frequencies (Hz)							
	1st Mode	2nd Mode	3rd Mode	4th Mode	5th Mode	6th Mode	7th Mode	8th Mode
Undamaged	9.189	9.222	16.201	23.595	23.954	27.951	29.895	38.703
50 %	9.264	9.296	16.209	23.769	24.085	28.067	29.887	38.786
75 %	9.301	9.333	16.212	23.855	24.149	28.125	29.883	38.826
100 %	9.338	9.370	16.216	23.940	24.213	28.183	29.878	38.867

**Table 3:** Accuracies of the CAE models corresponding to three levels of measurement errors for the multi-vehicle traffic loads

Damage Severity	Level of Measurement Error	Accuracy	FNR	FPR	Activation Function/Epoch	Final Training Loss	Threshold
Damage 100%	No error	82.1%	5.7%	12.2%	ReLU/5	1.08 × 10^−2^	1.09 × 10^−2^
	5% error	81.0%	7.1%	11.9%	ReLU/30	2.76 × 10^−3^	3.24 × 10^−3^
	10% error	81.0%	6.9%	12.1%	tanh/30	2.54 × 10^−3^	2.99 × 10^−3^
Damage 75%	No error	79.5%	8.3%	12.2%	tanh/10	3.96 × 10^−3^	4.35 × 10^−3^
	5% error	85.8%	5.3%	8.9%	ReLU/10	3.76 × 10^−3^	4.37 × 10^−3^
	10% error	82.3%	5.9%	11.8%	tanh/30	2.55 × 10^−3^	3.01 × 10^−3^
Damage 50%	No error	84.0%	5.4%	10.6%	ReLU/20	2.89 × 10^−3^	3.50 × 10^−3^
	5% error	73.3%	10.2%	16.5%	ReLU/20	3.16 × 10^−3^	3.43 × 10^−3^
	10% error	69.4%	10.3%	20.3%	tanh/20	2.73 × 10^−3^	2.80 × 10^−3^
	**Average**	**79.9%**	**7.2%**	**12.9%**			

**Table 4:** Accuracies of the CAE models corresponding to the three levels of measurement errors for the single-vehicle traffic load

Damage Severity	Level of Measurement Error	Accuracy	FNR	FPR	Activation Function/Epoch	Final Training Loss	Threshold
100%	No error	95.1%	0%	4.90%	tanh/30	1.35 × 10^−2^	1.41 × 10^−2^
	5% error	93.0%	0%	7.00%	tanh/85	4.23 × 10^−3^	5.07 × 10^−3^
	10% error	91.9%	0%	8.10%	tanh/50	9.28 × 10^−3^	9.76 × 10^−3^
75%	No error	92.6%	0.4%	7.00%	tanh/15	2.07 × 10^−2^	2.04 × 10^−2^
	5% error	90.1%	0.5%	9.40%	ReLU/60	9.56 × 10^−3^	9.70 × 10^−3^
	10% error	92.1%	0%	7.90%	tanh/250	1.32 × 10^−3^	1.92 × 10^−3^
50%	No error	91.9%	0.1%	8.00%	tanh/11	4.66 × 10^−2^	2.93 × 10^−2^
	5% error	92.5%	0%	7.50%	ReLU/75	5.09 × 10^−3^	6.29 × 10^−3^
	10% error	92.1%	0%	7.90%	tanh/55	6.92 × 10^−3^	7.58 × 10^−3^
	**Average**	92.4%	**0.1%**	**7.5%**			

**Table 5:** Comparison between the best accuracy threshold and approximate threshold.

**Type of Traffic Load**	**Damage Severity**	**Level of Measurement Error**	**For the Best Accuracy Threshold (the same as Table 3 and Table 4)**		**For Approximate Threshold**	
			**Threshold**	**Accuracy**	**Approximate Threshold (=3Q)**	**Adjusted Accuracy**
**Multi-vehicle traffic loads**	100%	No error	1.09 × 10^−2^	82.1%	1.09 × 10^−2^	82.0%
		5% error	3.24 × 10^−3^	81.0%	3.21 × 10^−3^	80.8%
		10% error	2.99 × 10^−3^	81.0%	2.97 × 10^−3^	81.0%
	75%	No error	4.35 × 10^−3^	79.5%	4.33 × 10^−3^	79.4%
		5% error	4.37 × 10^−3^	85.8%	4.17 × 10^−3^	84.9%
		10% error	3.01 × 10^−3^	82.3%	2.98 × 10^−3^	82.1%
	50%	No error	3.50 × 10^−3^	84.0%	3.37 × 10^−3^	83.7%
		5% error	3.43 × 10^−3^	73.3%	3.63 × 10^−3^	72.3%
		10% error	2.80 × 10^−3^	69.4%	3.15 × 10^−3^	67.6%
		**Average**		**79.8%**		**79.3%**
**Single-vehicle traffic load**	**Damage Severity**	**Level of Measurement Error**	**Threshold**	**Accuracy**	**Approximate Threshold (=3Q + 0.3IQR)**	**Adjusted Accuracy**
	100%	No error	1.41 × 10^−2^	95.1%	1.37 × 10^−2^	91.5%
		5% error	5.07 × 10^−3^	93.0%	4.93 × 10^−3^	91.4%
		10% error	9.76 × 10^−3^	91.9%	9.61 × 10^−3^	90.4%
	75%	No error	2.04 × 10^−2^	92.6%	2.02 × 10^−2^	90.5%
		5% error	9.70 × 10^−3^	90.1%	9.76 × 10^−3^	89.4%
		10% error	1.92 × 10^−3^	92.1%	1.81 × 10^−3^	90.5%
	50%	No error	2.93 × 10^−2^	91.9%	2.91 × 10^−2^	90.7%
		5% error	6.29 × 10^−3^	92.5%	6.14 × 10^−3^	90.5%
		10% error	7.58 × 10^−3^	92.1%	7.53 × 10^−3^	90.9%
		**Average**		**92.4%**		**90.6%**
